# Breaking Myths: The Underexplored Impact of the Ketogenic Diet on Managing Drug-Resistant Epilepsy in Infancy

**DOI:** 10.7759/cureus.77805

**Published:** 2025-01-22

**Authors:** Soma Basu, Hemamalini Arambakkam Janardhanam, Ranjith Kumar Manokaran

**Affiliations:** 1 Department of Clinical Nutrition, Faculty of Allied Health Sciences, Sri Ramachandra Institute of Higher Education and Research, Chennai, IND; 2 Department of Neurology, Sri Ramachandra Institute of Higher Education and Research, Chennai, IND

**Keywords:** drug-resistant epilepsy, genetic epilepsy, infancy, ketogenic diet, nutritional status, seizures

## Abstract

Introduction: Drug-resistant epilepsy (DRE) is a challenging neurological condition in infants. Breastfeeding is widely regarded as the gold standard for infant nutrition due to its immunological and developmental benefits. However, in certain cases of genetic DRE, breastfeeding has been associated with increased seizure frequency, necessitating alternative nutritional strategies. This study represents the first investigation that evaluates the efficacy of a milk-based ketogenic diet (KD) in infants aged one to six months with genetic DRE focusing on seizure control, nutritional status, developmental progress, and safety, as measured by metabolic and biochemical parameters. As the first study of its kind, this research offers a unique contribution to the field, paving the way for further investigations into diet-based therapies for refractory epilepsy in early life.

Methodology: This prospective study included eight infants with genetic DRE, aged one to six months. Baseline data on seizure frequency, nutritional status, developmental milestones, and biochemical parameters were collected. The KD was started at a 2:1 ratio based on a non-fasting KD protocol and was optimized based on need and tolerance. Nutritional status was assessed with the Strong Kids Nutrition Screening Tool. Seizure frequency was tracked daily, and urine ketone levels were monitored to confirm ketosis. Biochemical parameters were measured at baseline and after six months. Paired t-tests were used to analyze data.

Results: At baseline, the mean seizure frequency was 16.5/day. After six months on the KD, the mean seizure frequency decreased to 4.6/day (p < 0.001). Nutritional status improved significantly. Urine ketone levels remained consistently high. Biochemical parameters, including triglycerides and random blood sugar, showed no significant changes, confirming the diet's safety.

Conclusion: This study demonstrates that a milk-based KD is an effective and safe treatment for reducing seizures and improving nutritional and developmental outcomes in infants with genetic DRE. Persistent ketosis, indicated by large urine ketone levels, was a reliable biomarker of diet efficacy. Regular monitoring and careful parental counseling are essential for optimizing treatment outcomes in this vulnerable population. Further research with larger cohorts is needed to refine dietary protocols for infants with DRE.

## Introduction

Drug-resistant epilepsy (DRE) is a severe neurological disorder characterized by persistent seizures that fail to respond to at least two appropriately chosen and tolerated antiepileptic drugs. Infants with DRE represent a particularly vulnerable population due to their rapid neurodevelopmental trajectory and the limited availability of safe and effective therapeutic options. While the ketogenic diet (KD), a high-fat, low-carbohydrate therapeutic intervention, has demonstrated robust efficacy in reducing seizure frequency and improving neurological outcomes in older children, its application in infants remains significantly underexplored. This knowledge gap is critical given the unique physiological and metabolic considerations of infants [1,2]. Breastfeeding is widely regarded as the gold standard for infant nutrition due to its immunological and developmental benefits. However, in certain cases of genetic DRE, breastfeeding has been associated with increased seizure frequency, necessitating alternative nutritional strategies. Although the KD has shown promise as a non-pharmacological treatment for refractory epilepsy in older age groups, its implementation in infants, particularly in the form of a milk-based KD, has not been systematically studied [3,4]. This study represents the first investigation that evaluates the efficacy of a milk-based KD in infants aged 1-6 months with genetic DRE, focusing on seizure control, nutritional improvement, developmental progress, metabolic ketosis as measured by urine ketone levels, and safety as assessed through biochemical parameters, including triglyceride (TG) levels. By addressing this critical gap, our study provides novel insights into the potential role of KD in managing DRE in infants, a demographic for which therapeutic alternatives are scarce. As the first study of its kind, this research offers a unique contribution to the field, paving the way for further investigations into diet-based therapies for refractory epilepsy in early life.

## Materials and methods

Study design and population

A prospective study included eight infants aged one to six months diagnosed with genetic DRE. The study was conducted at Department of Neurology, Sri Ramachandra Hospital, Chennai, Tamil Nadu, India.

Ethical consideration

The study was approved by the Institutional Ethical Committee of Sri Ramachandra Institute of Higher Education and Research, Porur, Chennai. (REF NO: IEC/24MAR/185/09). 

Type of sampling and reasons for selection

The study was carried out for a duration of three months and included children aged one month to six months with DRE who were referred by the epileptologist for dietary intervention between June 2024 to November 2024. Eligible participants were referred by a Pediatric Epileptologist.

Patient consent statement

The inclusion of this study was in line with the Declaration of Helsinki. Parental consent was taken before enrolling the participants. All potential participants' parents were informed about the research goals. Parents were assured that no adverse effects would be anticipated from their child’s participation in the current research. Furthermore, their data were guaranteed to be anonymous and confidential, and their collaboration was voluntary. The participants were included in the study after getting the parental consent.

Inclusion criteria

Infants aged one to six months diagnosed with genetic DRE and non-amenable to epilepsy surgery were included.

Exclusion criteria

Infants with certain metabolic disorders such as defects in fatty acid oxidation, carnitine deficiency, and certain organic acidemias or those with a history of pancreatitis, pre-existing liver conditions, gastrointestinal disorders, and critically ill patients.

Data collection method

Baseline data on seizure frequency, nutritional status, developmental milestones, and biochemical parameters were collected. Parents initially expressed reluctance to wean their children off breastmilk. However, counseling sessions and observed increases in seizure activity linked to breastmilk intake facilitated a gradual transition to a milk-based KD. The milk-based KD constituted milkshake recipes using soya milk and almond milk, to the milk of various nuts, cereals, and pulses added in the required proportion to maintain the higher ketosis. The diet was initiated at a 2:1 ketogenic ratio and adjusted to 2.4:1 over the study period based on clinical response and tolerance. Nutritional status was assessed using the Strong Kids Nutrition Screening Tool, with scores categorized as poor (≥4), moderate (2-3), or good (0-1). A developmental assessment was done by a Pediatric Epileptologist. Seizure frequency was recorded daily by caregivers and validated by clinicians. Urine ketone levels were measured at multiple time points throughout the day to confirm sustained ketosis, with "large" ketone levels (>160 mg/dL) considered indicative of diet efficacy. Biochemical parameters, including random blood sugar (RBS), TG levels, renal profile, serum electrolytes, and complete blood count (CBC), were monitored at baseline and six months to ensure safety.

Statistical analyses

Data analysis was done using paired t-tests to compare baseline and endpoint outcomes, and descriptive statistics summarized seizure reduction categories.

## Results

**Figure 1:**
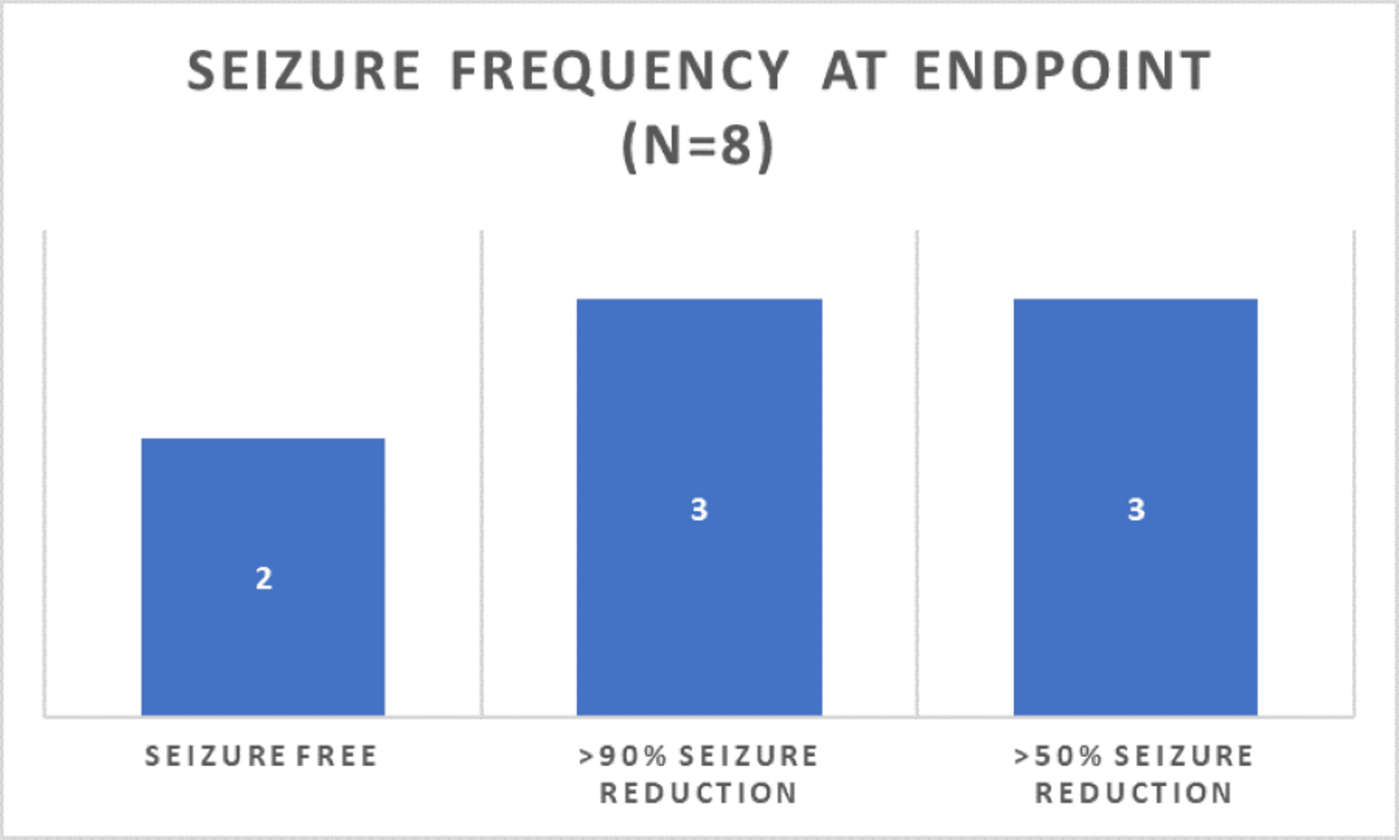
Seizure Frequency Following KD After six months of the ketogenic diet, seizure frequency significantly decreased, with two children (25%) becoming seizure-free, three (37.5%) achieving a ≥90% reduction, and three (37.5%) achieving a ≥50% reduction. KD: Ketogenic diet

Of eight eligible participants enrolled in the study, three infants (37.5%) were in the 1-3-month age group, while five (62.5%) were in the 4-6-month age group. The gender distribution comprised five male participants (62.5%) and three female participants (37.5%). All participants were diagnosed with genetic DRE (Table 1). At baseline, the median seizure frequency was 15/day (range: 10-25), with nutritional status categorized as poor in 62.5% (n=5) and moderate in 37.5% (n=3) of participants. After six months of the KD, seizure frequency significantly decreased, with two children (25%) becoming seizure-free, three (37.5%) achieving a ≥90% reduction, and three (37.5%) achieving a ≥50% reduction (Figure 1). The mean seizure frequency reduced from 16.5/day (SD: ±5.2) to 4.6/day (SD: ±3.8; p < 0.001).

**Table 1:** Descriptive Data Of eight eligible participants enrolled in the study three infants (37.5%) were in the 1–3-month age group, while five (62.5%) were in the 4–6-month age group. The gender distribution comprised five male participants (62.5%) and three female participants (37.5%).

Variable	Category	Number
Age (months)	1-3 months:	3 (37.5%)
	4-6 months	5 (62.5%)
Gender	Male	5 (62.5%)
	Female	3 (37.5%)

Nutritional status showed marked improvement, with all children achieving a "good" score (mean score reduced from 3.1 ± 0.6 to 0.4 ± 0.5; p < 0.001). Developmental progress was noted at the endpoint when compared with the baseline as assessed by the Pediatric Epileptologist. Urine ketone levels consistently measured "large" throughout the day, indicating robust metabolic ketosis and effective adherence to the KD.

Biochemical parameters remained within normal ranges at both baseline and six-month follow-up. RBS was stable (mean: 85.3 ± 6.2 mg/dL at baseline vs. 87.5 ± 5.8 mg/dL at endpoint). TG levels showed no significant elevation, with mean values at baseline of 92.5 ± 12.3 mg/dL compared to 96.1 ± 10.7 mg/dL at endpoint (p > 0.05). Renal profile, serum electrolytes, and CBC values also remained normal throughout the study period, indicating the diet's safety (Table 2). 

**Table 2:** Baseline and Endpoint Comparison of Key Parameters Baseline: at the time of enrollment; endpoint: after six months of ketogenic diet initiation; comparison of key parameters was done using mean, standard deviation (SD), the paired t-test, and the chi-square test.

Parameter	Baseline (During Enrollment) Mean ± SD	Endpoint (At sixth month) Mean ± SD	p-value
Strong Kids Score	3.1 ± 0.6	0.4 ± 0.5	< 0.001
Seizure Frequency (per day)	16.5 ± 5.2	4.6 ± 3.8	< 0.001
Urine Ketone Levels	Negative	Large (>160 mg/dL consistently)	-
Random Blood Sugar (mg/dL)	85.3 ± 6.2	87.5 ± 5.8	>0.05
Triglyceride (mg/dL)	92.5 ± 12.3	96.1 ± 10.7	>0.05

## Discussion

The results demonstrate the efficacy and safety of an individualized milk-based KD in managing genetic DRE among infants. Sustained ketosis, as evidenced by persistently high urine ketone levels, was associated with significant seizure reduction, improved nutritional status, and enhanced developmental outcomes. The maintenance of normal biochemical parameters, including RBS, TG levels, renal profile, serum electrolytes, and CBC, underscores the diet’s safety and tolerability in this vulnerable population [3,5].

The observation that TG levels remained within a normal range, despite the high-fat nature of the KD, is particularly noteworthy and alleviates concerns about potential hyperlipidemia in this age group. Initially, parental reluctance to wean breastmilk posed a challenge, but the clear correlation between breastfeeding and increased seizure frequency led to gradual acceptance of the ketogenic regimen. The transition to a KD was well-tolerated, with adjustments to a 2.4:1 ratio optimizing seizure control while maintaining nutritional adequacy.

These findings align with evidence from older children, supporting the role of KD as a non-pharmacological treatment for epilepsy [1,2]. However, the application in infants demands rigorous monitoring of metabolic, developmental, and biochemical parameters to prevent potential complications. Sustained ketosis, as reflected in large urine ketone levels, emerged as a critical biomarker of dietary efficacy, underscoring the importance of regular metabolic assessments.

Furthermore, a recent study demonstrated significant seizure reduction and cognitive improvement among children on a classical KD, reaffirming its potential in managing refractory epilepsy [6]. Similarly, another study found that a modified ketogenic approach resulted in improved seizure control and quality of life in children with intractable epilepsy, highlighting its adaptability across different age groups [7]. A systematic review emphasized the diet's ability to reduce seizure frequency without compromising growth or development, even in pediatric populations with severe epilepsy [8]. Recent evidence also indicates that KDs may have neuroprotective benefits, enhancing neuroplasticity and reducing oxidative stress in epileptic brains [9,10]. These results collectively reinforce the importance of individualized dietary interventions as adjunctive therapies in epilepsy management.

Limitations of the study

Despite its promising outcomes, the study has some limitations. First, the sample size was small, limiting the generalizability of the findings. Lastly, adherence to the KD requires careful monitoring and parental commitment, which may limit its widespread applicability.

Outcome of the study

The study demonstrated a significant reduction in seizure frequency, with two infants achieving complete seizure freedom and others showing substantial improvement. Nutritional status improved markedly, and developmental progress was observed in all participants. Sustained metabolic ketosis, as evidenced by consistent urine ketone levels, and stable biochemical parameters confirmed the diet's safety and tolerability. These outcomes highlight the potential of a milk-based KD to address the critical therapeutic needs of infants with DRE.

Rationale of the study

The rationale for this study was rooted in the unmet clinical need for effective and safe therapeutic options for infants with DRE, a particularly vulnerable population. Given the robust efficacy of the KD in older children and the absence of systematic studies in infants, this research aimed to bridge this knowledge gap. By focusing on a milk-based KD tailored for infants, the study sought to explore a novel intervention that aligns with the unique physiological and nutritional requirements of this age group, offering a potential breakthrough in the management of refractory epilepsy in early life.

## Conclusions

This study represents a pioneering effort to evaluate the efficacy and safety of a milk-based KD in infants aged 1-6 months diagnosed with genetic DRE. The findings demonstrate that the KD is highly effective in reducing seizure frequency, with 25% of participants becoming seizure-free and 75% achieving a significant reduction in seizures. Furthermore, the diet improved nutritional status and developmental progress without causing adverse metabolic effects. These results underscore the therapeutic potential of a milk-based KD as a viable non-pharmacological intervention for infants with DRE.
